# Osteoclastogenesis Behavior of Zirconia for Dental Implant

**DOI:** 10.3390/ma12050732

**Published:** 2019-03-04

**Authors:** Boldbayar Ganbold, Seong-Kyun Kim, Seong-Joo Heo, Jai-Young Koak, Zang Hee Lee, Jaejin Cho

**Affiliations:** 1 Department of Prosthodontics and Dental Research Institute, School of Dentistry, Seoul National University, 101 Daehak-ro, Jongno-gu, Seoul 03080, Korea; boldmonuud@yahoo.com; 2 Department of Prosthodontics and Dental Research Institute, Seoul National University Dental Hospital, School of Dentistry, Seoul National University, 101 Daehak-ro, Jongno-gu, Seoul 03080, Korea; 0504heo@snu.ac.kr (S.-J.H.); young21c@snu.ac.kr (J.-Y.K.); 3 Department of Cell and Developmental Biology, School of Dentistry, Seoul National University, 101 Daehak-ro, Jongno-gu, Seoul 03080, Korea; zang1959@hotmail.com; 4 Department of Dental Regenerative Biotechnology, School of Dentistry, Seoul National University, 101 Daehak-ro, Jongno-gu, Seoul 03080, Korea; jcho@snu.ac.kr

**Keywords:** zirconia, osteoclast, titanium, implant, surface

## Abstract

Zirconia is worth studying as an alternative to dental titanium implants to overcome the disadvantages of titanium. This study investigated the surface characteristics of the zirconia implant material and osteoclastogenesis responses on the surface compared with titanium. Yttrium oxide-stabilized 5% tetragonal zirconia polycrystalline specimens were manufactured, and osteoclast-precursor cells were cultured and differentiated into osteoclasts on the specimens. Surface shape, roughness, and chemical composition were evaluated. After culturing, cell morphologies and differentiation capacity were analyzed using tartrate-resistant acid phosphatase activity (TRACP). mRNA of two critical transcription factors, nuclear factor of activated T-cells 1 (*NFATc1*) and *c-Fos* were measured, and protein levels of *NFATc1* and *c-Fos* were investigated. The zirconia specimens had rhomboid-like shapes with smooth surfaces and exhibited no difference in surface roughness compared to the titanium specimens. Morphologies of differentiated osteoclasts on both materials were similar. TRACP activity on the zirconia showed comparable results to that on the titanium. The mRNA value of *NFATc1* on the zirconia was higher than that on the titanium at day four. The protein level of *c-Fos* was expressed thicker on the zirconia when compared to the titanium at day two. The results of this study suggest that zirconia material provides adequate osteoclastogenesis behaviors for dental implant use.

## 1. Introduction

Titanium (Ti) has been studied for years not only for dental implants but also for medical fields including orthopedic implants because of its great biocompatibility and high bone-tissue affinity [1,2,3]. In addition, modification of Ti material itself and Ti surface treatment have been experimented to obtain better properties [4,5,6,7,8]. Bernardi et al. [4] investigated the antibacterial properties of anatase-coated titanium healing screws and revealed that the coated screw preserved implant-surrounding tissues from micro-organism. Until now, Ti-based alloys are the most widely used as dental implant materials and exhibit good mechanical characteristics and long-term clinical success [9,10]. However, there are disadvantages to using Ti in dental applications. Clinicians have been forced to study hypersensitivity and allergic reactions in patients with dental implants due to unexplained implant failures [11]. Twenty-one of the 56 people who had received Ti implant showed a sensitive reaction to Ti through lymphocyte transformation tests [12]. An in vitro study investigated soft tissue discoloration placing Ti and ceramic materials under grafted mucosa [13], and Ti induced the most prominent discoloration in the mucosa.

The negative effects of Ti have driven many researchers to investigate the potential of ceramic implants for use in dental applications. Plecko et al. [14] compared four different metallic implant materials for their osseointegration and biocompatibility, and reported that zirconium and/or titanium based coatings improved osseointegration of cobalt–chrome and showed osseointegration similar to pure titanium [14]. Ceramic materials have been utilized in dental fields for long periods [15]. To overcome the drawbacks of Ti implants, ceramic material has been focus of research. Zirconium oxide, such as alumina-toughened zirconia and zirconia-toughened alumina, showed potential as an alternative to yttrium oxide-stabilized tetragonal zirconia polycrystals [15]. Strong ceramics exhibited bio-inert properties similar to stainless steel and showed a lower release of ions compared to metals [16]. Osseointegration between bone and ceramic material has been observed without inflammation, further supporting the potential use of ceramics in dental implants [17]. As a ceramic implant material, zirconia is composed of yttrium oxide (Y_2_O_3_)-stabilized 5% tetragonal zirconia polycrystals (Y-TZP) and is used in dental restorations [18]. Zirconia implants have the potential to replace Ti implants for short-term clinical use because both materials cause a similar hard tissue response [19,20]. Zirconia implants decreased bacterial adhesion compared to that of Ti implants, reducing the potential for inflammation around the implants [21]. Osteoblasts, a primary cell critical for bone growth and osseointegration, were able to attach to zirconia surfaces [22]. 

In perspective of bone physiology, cooperative interactions occur between osteoblasts and osteoclasts, which contribute to bone formation and resorption. And both cells are required for bone remodeling after implant insertion [23]. Bone necrosis of the jaw called antiresorptive agent-related osteonecrosis of the jaw could occur when a patient with osteoporosis uses an antiresorptive agent, a bisphosphonate which inhibits osteoclast activity of resorption [24,25,26]. This phenomenon indicates the balance between osteoblast and osteoclast is essential in bone metabolism [25,26]. Many researches on osseointegration of implant surfaces have focused on bone-forming osteoblasts. Much less is known regarding the function of osteoclasts in osseointegration. Osteoclasts are critical for bone resorption and have roles in early and late tissue responses after implant insertion [27].

We hypothesized that zirconia would be an acceptable material for osteoclast differentiation and have conducted experimental osteoclastogenesis on the surface of zirconia and Ti. The current study investigated the characteristics of zirconia compared to Ti surfaces assessing visual and quantitative properties of osteoclasts on zirconia and Ti disks. Two indicating factors for differentiation of osteoclasts in mRNA and protein level were examined to analyze gene expression.

## 2. Materials and Methods

### 2.1. Zirconia and Titanium Specimen Preparation and Analyses of Surface Characteristics

As the experimental group, thirty zirconia disks were fabricated via cold isostatic pressing at 200 MPa then sintered for 2 h at 1650 °C in air. Zirconia specimens were composed of yttria-stabilized tetragonal zirconia polycrystals (94.5 mol% ZrO_2_, 5.1 mol% Y_2_O_2_, 0.25 mol% Al_2_O_3_) (Ecucera, Pocheon, South Korea). Each disk had uniform dimensions with a 15-mm diameter and 1-mm thickness. Thirty machine-surfaced Ti disks (15 mm in diameter and 1 mm in thickness) were manufactured from commercially pure Ti grade IV (Warantec, Seoul, South Korea) as a control. All disks were washed in distilled water and ultrasonically cleaned with alcohol. Disks were then dried and autoclaved at 120 °C for 30 min. The disks surfaces were coated with a platinum film and viewed with a field emission scanning electron microscope (FE-SEM) (S-4700; Hitachi, Tokyo, Japan) using an acceleration voltage of 15 kV at three magnifications (×1000; ×5000; ×20,000). 3D images and surface roughness were obtained by confocal laser scanning microscopy (CLSM) (LSM 5; Pascal, Zeiss, Obercochen, Germany). Energy dispersive X-ray spectroscopy (EDS) (EX-250; Horiba, Tokyo, Japan) was conducted to assess the chemical composition of the disks. Each evaluation was conducted with three disks for each group.

### 2.2. Analyses of Osteoclastogenesis on Zirconia and Titanium Specimens

#### 2.2.1. Raw Cell Preparation and Osteoclast Differentiation Processing

Murine RAW264.7 macrophage/monocyte cells (TIB-71; American Type Culture Collection, Rockville, MD, USA) were cultured on the surfaces of zirconia and Ti disks [28]. Disks were placed at the bottom of a 12-well plate at 37 °C in a humidified atmosphere of 5% CO_2_ in Minimum Essential Medium Alpha (α–MEM; Gibco, Grand Island, NY, USA) containing 10% fetal bovine serum (FBS; Gibco, Rockville, MD, USA), 100 U/mL penicillin, and 100 µg/mL streptomycin. The cells were suspended in 250 µL α–MEM, and were directly seeded on top of the disk surface at a density of 2 × 10^4^ cells/disk, not on the residual space of the well. After adhering cells to the disk for 6 h, the medium was changed to cover the entire well [28]. One hundred nanograms/milliliter of mouse Receptor activator of nuclear factor Kappa-B ligand (RANKL, Peprotech, Rocky Hill, NJ, USA) was added to induce osteoclast differentiation. RANKL and medium were changed every two days.

#### 2.2.2. Osteoclast Cell Morphologic Analysis by FE-SEM

After three days of culture on the disks, cells were fixed with 2% glutaraldehyde in phosphate buffered saline (PBS) for 10 min at room temperature [28]. Cells were stained with 2% osmium tetroxide in PBS for 15 min and dehydrated in an ascending ethanol series. After critical point drying, samples were sputter-coated with 6 nm platinum and then examined in the FE-SEM. Observations were performed at three different areas on 4 disks for each group.

#### 2.2.3. Tartrate-Resistant Acid Phosphatase Activity Assay

Tartrate-resistant acid phosphatase (TRACP) enzyme activity was measured to quantitatively evaluate osteoclast differentiation activity. Cells were treated with RANKL for three and five days, and TRACP activity was measured using the TRACP assay kit (Takara, Kyoto, Japan) according to the manufacturer’s protocol. The TRACP activities of the cultured cells were examined by a colorimetric assay utilizing the conversion of colorless p-nitrophenol phosphate to colored p-nitrophenol. The concentration of p-nitrophenol was measured spectrophotometrically by taking sample absorbance readings at 405 nm (Bio-Rad, Hercules, CA, USA). Replicate measurements were performed with 4 disks for each group.

#### 2.2.4. Osteoclast Gene Analysis by mRNA Expression Assessment

Real-time reverse transcriptase-polymerase chain reaction (RT-PCR) was conducted to investigate mRNA levels in osteoclast cells for one reference gene, *glyceraldehyde 3-phosphate dehydrogenase* (*GAPDH*), and two target genes, nuclear factor of activated T-cells 1 (*NFATc1*) and *c-Fos*. Total ribonucleic acid (RNA) was isolated from cell samples after two and four days of treatment with RANKL. Cells were lysed by adding the TRIzol^®^ reagent (Invitrogen, Carlsbad, CA, USA) according to the manufacturer’s instructions. The homogenized samples were incubated for 5 min at room temperature to permit the complete dissociation of nucleoprotein complexes. Then, 0.2 mL of chloroform was added to each sample, and the samples were mixed vigorously and then incubated at room temperature for 3 min. The samples were centrifuged at 12,000× *g* for 15 min at 4 °C. Following centrifugation, the mixture was separated into a lower red, phenol-chloroform phase, an interphase, and a colorless upper aqueous phase. RNA was precipitated from the aqueous phase by mixing with 0.4 mL of isopropyl alcohol. The samples were incubated at room temperature for 10 min and centrifuged for 10 min at 4 °C. The RNA precipitate formed a gel-like pellet on the side and bottom of the tube. After removing the supernatant, the RNA pellet was washed with 1 mL of 75% ethanol and centrifuged at 12,000× *g* for 5 min at 4 °C. The RNA pellet was dried for 5 min. RNA was dissolved in RNase-free water and incubated at 55 °C for 10 min. Total RNA was quantified using a Nanodrop Spectrophotometer (ThermoScientific Nanodrop Technologies, Wilmington, DE, USA); 1 μg of total RNA was reverse transcribed to cDNA at 42 °C for 50 min using the Superscript II (SSII) reverse transcriptase (Invitrogen) that contains oligo (dT), 2.5 mM dNTP, 5xFS buffer, 0.1M DTT, SSII enzyme, and RNase out.

Real-time PCR was performed in an iCycler (Bio-Rad) using SYBR green (Invitrogen) detection. Each reaction contained 5 μL of cDNA, 0.4 μL of the forward and reverse specific primers (Table 1), and 0.4 μL of rhodamine X (ROX) in a final volume of 20 μL. The amplification program consisted of a pre-incubation step for denaturation of the template cDNA (3 min 95 °C), followed by 40 cycles consisting of a denaturation step (15 s for 95 °C), an annealing step (15 s for 60 °C), and an extension step (30 s for 72 °C). Samples were run in triplicate (n = 12). Levels of *c-Fos* and *NFAcT1* were normalized to *GAPDH*.

#### 2.2.5. Osteoclast Gene Analysis by Protein Expression Assessment

The protein expression levels of 2 genes, *c-Fos*, and *NFATc1*, were assessed via Western blot analysis [29]. RAW264.7 cells were grown on Ti and zirconia disks for one, two, and three days after treatment of RANKL. After washing with PBS, total cell lysates were harvested by lysing the cells with chilled radioimmunoprecipitation assay (RIPA) buffer (10 mM Tris pH 7.2, 150 mM NaCl, 5 mM ethylenediaminetetraacetic acid, 1 mM NaF, 2.5 mM sodium pyrophosphate, 1 mM sodium orthovanadate, 1 mM phenylmethylsulfonyl fluoride, 1 μg/mL leupeptin, 1 µg/mL aprotinin, 1% Triton X-100, 0.1% SDS and 1% deoxycholate). Total cell lysates were incubated for 20 min and centrifuged at 14,000× *g* for 15 min at 4 °C. Harvested proteins were separated by 8% sodium dodecyl sulfate-polyacrylamide gel electrophoresis (SDS-PAGE) and transferred to a nitrocellulose membrane (Whatman GmbH, Dassel, Germany). The membrane was probed with specific antibodies (Santa Cruz Biotechnology, Heidelberg, Germany) with a concentration of 2 μg/mL and the reactivity of the immune complexes were detected by using enhanced chemiluminescence (ECL, GE Healthcare, Hatfield, Hertfordshire, UK) reagents.

### 2.3. Statistical Analysis

Statistical significance for all data comparisons was determined by student *t*-test analysis (SPSS 23, IBM Inc., Armonk, NY, USA). *p*-values less than 0.05 were considered statistically significant.

## 3. Results

### 3.1. Surface Characteristics of the Zirconia and the Titanium Specimens

**Figure 1:**
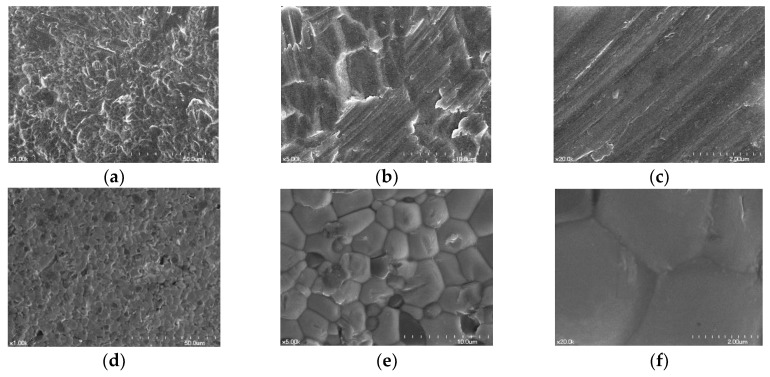
Scanning electron microscope (SEM) images of the surfaces of Ti disk and zirconia disk; Control group = Ti disk; each row, left to right, original magnification (**a**) 1000×; (**b**) 5000×; (**c**) 20,000×. Test group = zirconia disk: (**d**) 1000×; (**e**) 5000×; (**f**) 20,000×.

In SEM views, the zirconia specimens had rhomboid-like shapes with smooth surfaces, and the Ti specimens exhibited a flat surface with striped patterns. (Figure 1).

**Figure 2:**
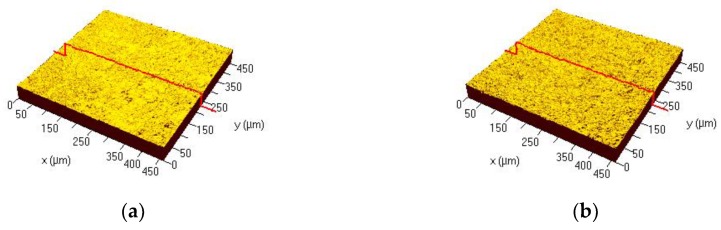
3D images of the surface microstructures with dimensional range of 50 µm as captured by confocal laser scanning microscopy (CLSM). (**a**) Control group = Ti disk; (**b**) Test group = zirconia disk.

**Figure 3:**
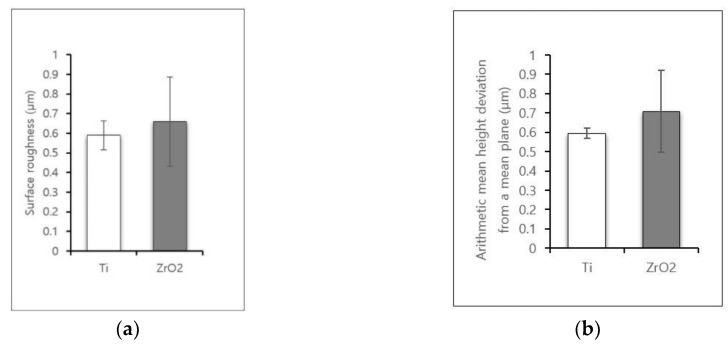
Surface roughness-related parameters obtained by CLSM. (**a**) Ra (surface roughness); (**b**) Sa (arithmetic mean height deviation from a mean plane.

3D image of the microstructures on the surface of each disk is shown in Figure 2. Results for the surface roughness-related parameters for each disk are summarized in Figure 3. The Ra (surface roughness) and Sa (arithmetic mean height deviation from a mean plane) values for Ti disks were 0.59 ± 0.07 µm and 0.59 ± 0.02 µm, respectively. The Ra and Sa values for zirconia disks were 0.66 ± 0.22 µm and 0.71 ± 0.21 µm, respectively. There was no statistically significant difference between the overall roughness of Ti and zirconia surfaces. 

**Figure 4:**
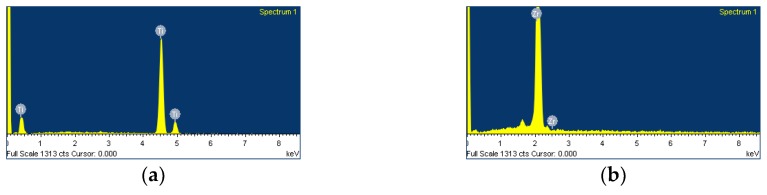
Energy dispersive X-ray spectroscopy (EDS) patterns of the zirconia disk compared with the Ti disk. (**a**) Control group = Ti disk; (**b**) Test group = zirconia disk.

EDS spectra revealed Zi peaks for zirconia disks and Ti peaks for Ti disks, confirming that both disks were pure and free from any contamination for further cell experiments (Figure 4). 

### 3.2. FE-SEM Assessment of Cell Morphology

**Figure 5:**
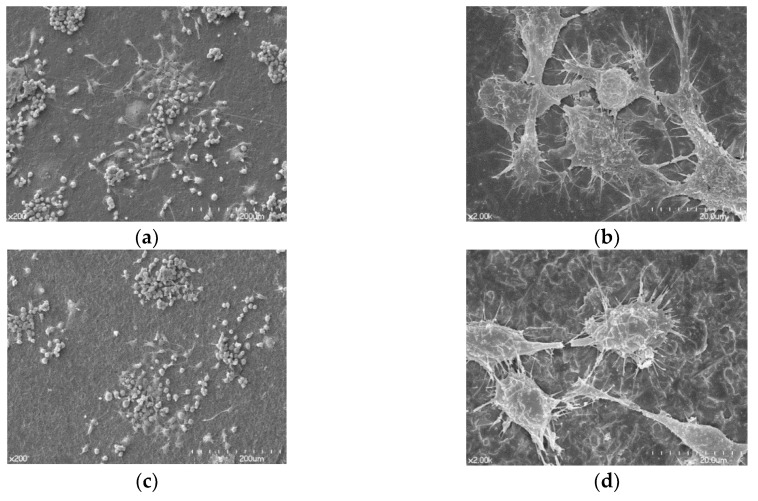
Scanning electron microscopy images showing differentiated osteoclasts that were seeded on the Ti disk and the zirconia disk. (**a**) Cells seeded on a Ti disk, original magnification 200×; (**b**) original magnification 2000×; (**c**) Cells seeded on a zirconia disk, original magnification 200×; (**d**) original magnification 2000×.

Differentiated osteoclasts morphologies are shown in Figure 5. Osteoclasts were spread regularly on the surfaces of both Ti and zirconia disks. A high magnification FE-SEM image (×2000) confirmed that cells grown on both surfaces displayed morphologies characteristic of well-differentiated osteoclasts. Osteoclasts on both Ti and zirconia disks showed similar morphologies that had irregular shapes with ruffled borders stretching out from its body, attached to the surface (Figure 5b,d).

### 3.3. Tartrate-Resistant Acid Phosphatase Activity

**Figure 6:**
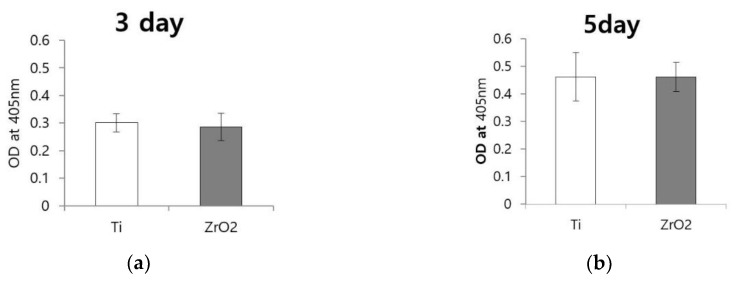
Tartrate-resistant acid phosphatase (TRACP) activity of osteoclasts on Ti disks and zirconia disks. (**a**) Absorbance values after three days of cell culturing; there was no significant difference between the two groups; (**b**) Absorbance value after five days of cell culture; there was no significant difference between the two groups. (*p* > 0.05; the bar represents mean ± SD).

At day three, absorbance value was 0.301 ± 0.016 for Ti and 0.286 ± 0.024 for zirconia disks (Figure 6a). At day five, absorbance value was 0.461 ± 0.043 for Ti and 0.461 ± 0.026 for zirconia disks (Figure 6b). There were no significant differences between the TRACP activities of cells grown on zirconia and Ti disks at either three or five days.

3.4. mRNA Expression of c-Fos and NFATc1

**Figure 7:**
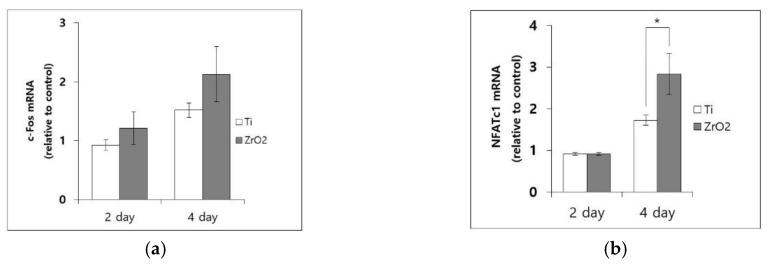
The mRNA levels of osteoclastogenic transcriptional factors on Ti disks and zirconia disks were measured by real time reverse transcriptase-polymerase chain reaction (RT-PCR). (**a**) *c-Fos* mRNA levels at day two and four; (**b**) *NFATc1* mRNA levels at day two and four. (* *p* < 0.05; the bar represents mean ± SD; all values are normalized to mRNA levels on Ti at day two).

mRNA levels assessed by RT-PCR on RNA harvested from cells that were treated with RANKL for two and four days are shown in Figure 7. All mRNA levels were normalized to values collected for Ti disks on day two. At day two, the relative expression of *c-Fos* mRNA was 1.205 ± 0.278 for zirconia and 0.921 ± 0.093 for Ti. At day four, the relative expression of *c-Fos* was 2.124 ± 0.474 for zirconia and 1.514 ± 0.124 for Ti. These results did not show any significant differences (Figure 7a). At day two, the relative expression of *NFATc1* mRNA was 0.913 ± 0.036 for zirconia and 0.911 ± 0.106 for Ti, which showed no significant difference. However, at day four, relative expression of *NFATc1* mRNA levels on zirconia (2.831 ± 0.492) was significantly higher than that on Ti (1.722 ± 0.158) (*p* = 0.02) (Figure 7b).

### 3.5. Protein Expression of c-Fos and NFATc1

**Figure 8:**
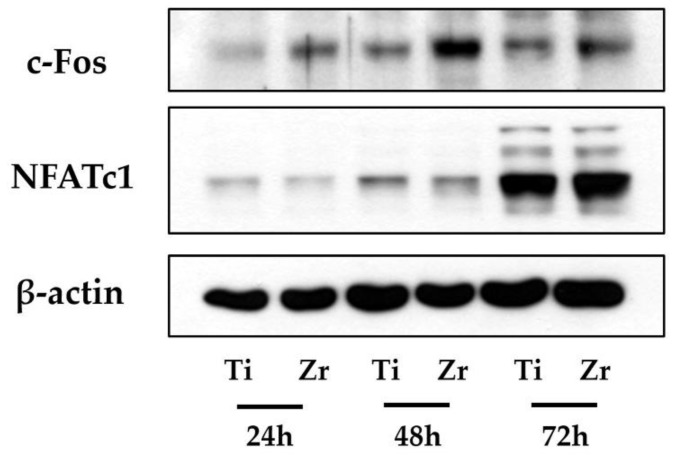
Protein levels of osteoclastogenic transcription factors in cells grown on Ti disks and zirconia disks were assayed by western blot analysis. Protein levels *c-Fos* on zirconia disks were thicker than Ti disks at day two. Protein levels of *NFATc1* were not different across Ti and zirconia disks at any time point.

Figure 8 shows protein expressions with western blot analysis for *c-Fos* and *NFATc1*. We observed that *c-Fos* protein levels were slightly thicker for zirconia disks when compared to Ti disks at day two whereas protein levels on zirconia and Ti disks were similar at day three. Protein levels of *NFATc1* were similar across all treatment groups.

## 4. Discussion

Osseointegration describes the structural and functional complex that occurs between an implant surface and the inner bone [30] which is critical for the survival of an implant after insertion into the body. For many decades, Ti has been used as a material for dental implants. However, the disadvantages of Ti as implant material have pushed dental clinicians to study other materials that could replace Ti. In the biomaterials field, ceramic materials are gaining popularity due to their increased strength, enhanced fracture resistance, durability, and excellent biocompatibility [31]. Zirconia is a polymorphic material that appears in three forms: monoclinic, cubic, and tetragonal and which is a novel material for application in dental ceramics [32]. This material has a high potential for use in dental implants due to its tooth-like color, its good mechanical properties, and its excellent biocompatibility [33]. It is possible that zirconia could be used as a non-metal implant and replace the use of titanium [34]. 

The present study may be the first study to compare osteoclast cell responses on zirconia and Ti materials directly. Given that osteoclast differentiation is likely to play an important role in peri-implant bone remodeling [35] we selected osteoclast cells as an experimental model, and evaluated diverse responses of osteoclasts on the zirconia surface. Mature osteoclasts can be induced to resorb bone upon stimulation with RANKL and macrophage-colony stimulating factor (M-CSF) [36]. Murine bone marrow macrophage cell line, RAW264.7 cells were used as osteoclast precursor cells. In this study, cells in 250 µL media were directly seeded on top of the disk surface with caution, not on the residual space of the well. Cells were incubated for 6 h to attach to the disk, and then the medium was changed to cover the entire well. Even though time was given for those to be attached on the disk, cells might possibly move to residual space of the well when they grew. If most cells grew on the disk, but some of the cells grew in the remaining space outside the disk, it might affect the results of this study. Nevertheless, since all the specimens were of the same size, there appears to be of value as a comparative study of the two materials.

To induce osteoclast differentiation, we allowed them to proliferate for 6 h and then treated them with RANKL. The effects of zirconia surface itself on osteoclast cellular responses were compared with those on Ti surface. In this study, a surface roughness of the zirconia disk did not show any difference with that of the Ti disk. Considering that the surface topography of materials contributed to differences in osteoclast behavior in previous reports [37,38,39], the surface roughness did not affect cellular responses in a comparison between the zirconia and Ti surfaces. We also showed that zirconia and Ti disks were pure and contained no contaminants using EDS. 

Potent TRACP activity is found in osteoclasts and is a suitable biochemical probe for osteoclast function [40,41]. TRACP is a lysosomal acid phosphatase that osteoclasts secrete to perform their specialized resorption process. TRACP activities of osteoclasts on Ti and zirconia disks for three and five days did not show any significant difference. Secreting similar amount of TRACP enzymes on both zirconia and Ti disks indicated that the quantities of differentiated cells on both disks were similar amounts. Moreover, FE-SEM displayed the same morphologies of osteoclasts on both Ti and zirconia disks. From quantitative and visual aspects, osteoclast behaviors on both disks were proven to be comparable to each other.

*c-Fos* and *NFATc1* are known as major transcription factors required for the osteoclast differentiation of terminal stage and are strongly induced by RANKL stimulation [42]. Western blot analysis was conducted to measure protein levels of *NFATc1* and *c-Fos*. Protein levels were assessed at one, two and, three days after treatment with RANKL. At day two, cells grown on zirconia showed higher levels of *c-Fos* protein relative to those grown on Ti. However, on day three, the abundance of *c-Fos* protein on both disks did not vary each other. Even though *c-Fos* is an important indicating factor for osteoclastogenesis, *NFATc1* could induce osteoclast differentiation in the absence of *c-Fos* [43]. In other words, as long as the protein levels of *NFATc1* are similar on both disks, it could induce comparable osteoclast differentiation even when there is no *c-Fos* protein. In the case of *NFATc1*, there were no significant differences in protein levels between cells grown on zirconia and Ti disks for any experimental time point. These observations indicate that zirconia induces similar osteoclast cell responses relative to Ti. 

We conducted real-time RT-PCR to assess mRNA level of *NFATc1* and *c-fos* after treatment with RANKL for two and four days. At day two, there were no differences in mRNA levels of either transcription factors for cells grown on either Ti or zirconia disks. There was not a significant difference in *c-Fos* mRNA level between zirconia and Ti disks at day four. mRNA level of *NFATc1* of zirconia for day four was significantly higher than that of Ti. Annette et al. [44] reported poor correlations between mRNA transcript levels and the protein produced. Protein is the final product of transcription that proceeds direct modulation of osteoclastgenesis. This implies that variation in mRNA level may have little impact compared to the respective protein [44]. Taken together, the results of this study indicate that both disks displayed identical surface responses toward osteoclast behaviors.

Nonmetallic materials with high mechanical properties are required [15]. Low elasticity and mechanical property are the disadvantages of the zirconia implant [45,46]. There have been attempts to overcome its disadvantage, using polyetheretherketone restoration with zirconia implant [45], and manufacturing hybrid-type zirconia composing Ti [46]. Even with the current strength, zirconia implants may be appropriate for restoring anterior teeth to achieve aesthetic effects. However, when modification of the abutment angle is required, zirconia implants are difficult to use due to the one-piece manufacturing [15]. Additional research is needed to enable the fabrication of non-metallic implants with biomaterials, such as zirconia, through a digital workflow that enables custom-made, maneuverable, and well-adapted implants. As shown in the results, zirconia, featured similar osteoclast cell behaviors to Ti, and zirconia showed potential properties as a reliable implant material in terms of bioengineering perspectives. In the future, studies focusing not only on osteoclast behaviors but also on interactions with osteoblasts are helpful, and long-term in vivo studies regarding the direct effects of zirconia implants on osseointegration in comparison with Ti implants are needed before a robust recommendation of zirconia for the dental implant material. 

## 5. Conclusions

This study investigated surface characteristics of zirconia implants and osteoclast behaviors in gene level on the surface. Visual evaluation of differentiated osteoclasts confirmed that osteoclastogenesis for both zirconia and Ti implant disks were comparable. Given that two transcriptional factors appearing similarly on both disks, it is possible that zirconia implants could function like Ti implants and induce favorable osseointegration.

## Figures and Tables

**Table 1:** Genes and primer sequences used in reverse transcriptase-polymerase chain reaction (RT-PCR).

Gene Name	Primer Sequences (F = forward; R = reverse)
*GAPDH*	(F) 5′-TGCACCACCAACTGCTTAGC-3′, (R) 5′-GGCATGGACTGTGGTCATGAG-3′;
*c-Fos*	(F) 5′-CTGGTGCAGCCCACTCTGGTC-3′, (R) 5′-CTTTCAGCAGATTGGCAATCTC-3′;
*NFATc1*	(F) 5′-CGGCTGCCTTCCGTCTCATAG-3′, (R) 5′-CGGCTGCCTTCCGTCTCATAG-3′.
